# Transcriptomic Analysis of *Streptococcus suis* in Response to Ferrous Iron and Cobalt Toxicity

**DOI:** 10.3390/genes11091035

**Published:** 2020-09-02

**Authors:** Mengdie Jia, Man Wei, Yunzeng Zhang, Chengkun Zheng

**Affiliations:** 1 Joint International Research Laboratory of Agriculture and Agri-Product Safety, The Ministry of Education of China, Yangzhou University, Yangzhou 225009, China; jiamengdie@163.com (M.J.); fhzxmwei@163.com (M.W.); yzzhang@yzu.edu.cn (Y.Z.); 2 Jiangsu Key Laboratory of Zoonosis, Yangzhou University, Yangzhou 225009, China

**Keywords:** *Streptococcus suis*, transcriptome, ferrous iron, cobalt, RNA sequencing

## Abstract

*Streptococcus suis* is a zoonotic pathogen causing serious infections in swine and humans. Although metals are essential for life, excess amounts of metals are toxic to bacteria. Transcriptome-level data of the mechanisms for resistance to metal toxicity in *S. suis* are available for no metals other than zinc. Herein, we explored the transcriptome-level changes in *S. suis* in response to ferrous iron and cobalt toxicity by RNA sequencing. Many genes were differentially expressed in the presence of excess ferrous iron and cobalt. Most genes in response to cobalt toxicity showed the same expression trends as those in response to ferrous iron toxicity. qRT-PCR analysis of the selected genes confirmed the accuracy of RNA sequencing results. Bioinformatic analysis of the differentially expressed genes indicated that ferrous iron and cobalt have similar effects on the cellular processes of *S. suis*. Ferrous iron treatment resulted in down-regulation of several oxidative stress tolerance-related genes and up-regulation of the genes in an amino acid ABC transporter operon. Expression of several genes in the arginine deiminase system was down-regulated after ferrous iron and cobalt treatment. Collectively, our results suggested that *S. suis* alters the expression of multiple genes to respond to ferrous iron and cobalt toxicity.

## 1. Introduction

*Streptococcus suis* causes meningitis, septicemia, pneumonia, endocarditis, and arthritis in pigs, leading to major economic losses worldwide [1]. It also can be transmitted to humans by skin lesions or via the gastrointestinal tract, and is responsible for serious diseases such as meningitis and streptococcal toxic shock-like syndrome [2,3]. Currently, *S. suis* is divided into 29 serotypes based on capsular polysaccharide [4]. Among them, serotype 2 is the most prevalent serotype involved in both human and swine infections in most countries [5]. *S. suis* caused more than 1600 human deaths worldwide by 2013 [5]. In China, two large outbreaks of *S. suis* infection in humans occurred in 1998 and 2005, leading to 240 cases with 53 deaths in total [6]. More recently, sporadic cases of human infection of *S. suis* have been reported worldwide [7,8,9,10]. Moreover, it was the most prevalent bacterial pathogen in Chinese pig farms from 2013 to 2017 [11]. Thus, *S. suis* remains a continued threat to public health and to the swine industry.

Transition metals, such as iron and manganese, are essential nutrients for almost all organisms; many proteins require metals as cofactors to exert their biological activity [12]. The host can restrict the availability of metals to control bacterial infection [13]. Therefore, bacteria have evolved several mechanisms, such as using metal transporters, to acquire metals from various sources [13,14]. Although metals are important to bacteria, excess metals are toxic [15]. Imposition of metal toxicity is another strategy used by the host to respond to bacterial infection [14,15]. As a countermeasure, bacteria adopt metal efflux systems or other mechanisms to maintain metal homeostasis [15,16]. Metal homeostasis plays an essential role in bacterial physiology and pathogenesis [15]. For example, manganese homeostasis mediated by the manganese efflux pump (MntE) is critical for oxidative stress resistance and virulence in *Streptococcus pyogenes* and *Staphylococcus aureus* [17,18], while copper homeostasis conferred by MctB is essential for the virulence of *Mycobacterium tuberculosis* [19].

The mechanisms to maintain metal homeostasis in *S. suis* have been partly elucidated. Zur, a zinc uptake regulator, is involved in zinc homeostasis [20], while manganese homeostasis is mediated by MntE, a cation efflux family protein [21]. MntE also contributes to the oxidative stress response and virulence in *S. suis* [21]. The *S. suis* genome encodes a copper efflux system (CopA), which protects the bacterium against copper toxicity [22]. The genetic organization of *copA* in *S. suis* is different from that in related *Streptococci* species, in which the gene is a component of the Cu-responsive operon [22,23,24,25]. While these systems are specific to one metal, PmtA contributes to both ferrous iron and cobalt homeostasis [26]. Except for zinc, the metal homeostasis mechanisms for other metals have not been investigated from the transcriptome-level in *S. suis* [20,21,22,26].

In this study, the transcription profiles of *S. suis* grown in liquid medium supplemented with either ferrous iron or cobalt were compared with those after supplementation with deionized water. The data revealed that the expression of numerous genes was altered in response to ferrous iron and cobalt toxicity. Moreover, most of the genes differentially expressed in response to cobalt toxicity are the same as those expressed in response to ferrous iron toxicity.

## 2. Materials and Methods

### 2.1. Bacterial Strain and Growth Conditions

The *S. suis* 2 strain SC19 was isolated from the brain of a diseased pig during the 2005 outbreak in Sichuan province, China [27]. *S. suis* was grown at 37 °C in Tryptic Soy Broth (TSB) or on Tryptic Soy Agar (TSA, Becton, Dickinson and Company, Suzhou, China) supplemented with 10% (vol/vol) newborn bovine serum (Sijiqing, Hangzhou, China). Analytical-grade FeSO_4_ and CoSO_4_ salts were purchased from Sinopharm Chemical Reagent Co. Ltd. (Shanghai, China). CoSO_4_ stock (0.25 M) was prepared in deionized water and stored at room temperature. Since ferrous iron rapidly oxidizes to ferric iron, 2 M FeSO_4_ solution was prepared in deionized water before each use. Overnight culture of the SC19 strain was diluted 1:100 in fresh medium to achieve an initial OD_600_ of 0.03, and grown to the mid-exponential phase (OD_600_ = 0.6). The culture was then divided into 3 aliquots, which were supplemented with 2 mM FeSO_4_, 0.25 mM CoSO_4_, or deionized water. After 15 min of treatment, bacterial cells were collected by centrifugation for 1 min. The cell pellets were immediately used for RNA extraction. Three independent experiments were performed to obtain biological triplicate samples for each treatment.

### 2.2. RNA Extraction

RNA was extracted using an Eastep Super Total RNA Isolation Kit (Promega, Shanghai, China), according to the manufacturer’s protocol. During RNA extraction, DNase I digestion was performed to remove genomic DNA. RNA integrity and concentration were preliminarily evaluated by gel electrophoresis and spectrophotometric analysis on the Nanodrop 200 instrument (Allsheng, Hangzhou, China), respectively. Before cDNA library preparation and qRT-PCR, RNA concentration was further measured using the Qubit RNA Assay Kit in a Qubit 2.0 Flurometer (Life Technologies, Carlsbad, CA, USA), and RNA integrity was further assessed using the RNA Nano 6000 Assay Kit of the Bioanalyzer 2100 system (Agilent Technologies, Santa Clara, CA, USA). RNA samples with an RNA integrity number (RIN) above nine were used for cDNA library preparation and qRT-PCR.

### 2.3. cDNA Library Preparation and Sequencing

cDNA library preparation and sequencing were performed with the assistance of Novogene Bioinformatics Technology Co., Ltd. (Beijing, China). For cDNA library preparation, 3 µg of total RNA per sample was used as input material. The ribosomal RNA (rRNA) was removed using the Ribo-Zero rRNA Removal Kit (for bacteria) (Illumina, San Diego, CA, USA). Nine sequencing libraries, i.e., three treatments with three biological replications, were constructed using NEBNext Ultra Directional RNA Library Prep Kit for Illumina (NEB, Ipswich, MA, USA), according to the manufacturer’s recommendations. The library fragments were purified with AMPure XP system (Beckman Coulter, Beverly, MA, USA) to preferentially select cDNA fragments of 150~200 bp. Library quality was evaluated using the Agilent Bioanalyzer 2100 system (Agilent Technologies, Santa Clara, CA, USA). The qualified libraries were sequenced using the Illumina Hiseq^TM^ 4000 platform (Illumina, San Diego, CA, USA) and paired-end reads were generated.

### 2.4. RNA Sequencing Data Analysis

After removing reads containing the adapter and low-quality reads, clean reads were obtained, and the Q20 and Q30 values of the clean reads were calculated. The clean reads were mapped to the *S. suis* SC84 genome (GenBank accession number: NC_012924.1) by using Bowtie2-2.2.3 [28]. The FPKM (the expected number of fragments per kilobase of transcript sequence per million base pairs sequenced) method was used for quantification of gene expression levels [29]. DESeq R package (1.18.0) was used to identify differentially expressed genes (DEGs) between the two groups [30]. Genes with a fold change > 2 and an adjusted *p*-value  <  0.05 were defined as DEGs. The DEGs were subjected to GO enrichment analysis using the GOseq method, which is based on Wallenius non-central hyper-geometric distribution [31]. KOBAS 2.0 software with the hyper-geometric test was used for KEGG pathway enrichment analysis [32]. GO terms and KEGG pathways with a corrected *p*-value (*q* value) < 0.05 were considered to be significantly enriched.

### 2.5. Gene Expression Analysis by qRT-PCR

The RNA samples used for qRT-PCR analysis were the same as those used for RNA Sequencing. Approximately 500 ng RNA per sample was used to synthesize cDNA with the PrimeScript RT Reagent Kit with gDNA Eraser (TaKaRa, Dalian, China). Quantitative PCR was performed on a StepOnePlus Real-Time PCR System (Applied Biosystems, Waltham, MA, USA) using the NovoStart SYBR qPCR SuperMix Plus kit (novoprotein, Shanghai, China). The procedure for quantitative PCR was 95 °C for 1 min, followed by 40 cycles of 95 °C for 20 s, and 60 °C for 1 min. The experiments were performed with three biological replications and three technical replications. The relative gene expression level was analyzed using the 2^−ΔΔCT^ method [33], with 16S rRNA serving as the reference gene. Statistical analysis using the unpaired Student’s t test was performed to determine whether the differences were significant (*p* < 0.05). The primers used for qRT-PCR analysis are listed in Table 1. The efficiency of each primer pair was detected using serially diluted genomic DNA, as previously described [34], and was shown in Table S1.

## 3. Results

### 3.1. RNA Sequencing Information

To better understand the mechanisms of *S. suis* in response to ferrous iron and cobalt toxicity, the transcription profiles of *S. suis* following treatment with ferrous iron and cobalt were compared with that treatment with water by RNA sequencing analysis. The experiment was performed in three conditions, i.e., ferrous iron treatment (Fe), cobalt treatment (Co), and water treatment (control, Nor), with three biological replications for each condition. Illumina HiSeq sequencing of the nine libraries generated 9–13 million raw reads per library (Table 2). After removing reads containing adapter and low-quality reads, 8–13 million clean reads were retained for each library (Table 2). Approximately 98% of the Q20 value and 94% of the Q30 value were observed from RNA sequencing data (Table 2). More than 98% of the clean reads could be uniquely mapped to the reference genome of *S. suis* (Table 2), positive strand and negative strand approximately for each half. The RNA sequencing data have been deposited into the NCBI Gene Expression Omnibus (GEO), under accession number GSE153766.

### 3.2. Changes in S. suis Transcriptome in Response to Ferrous Iron and Cobalt Toxicity

**Figure 1:**
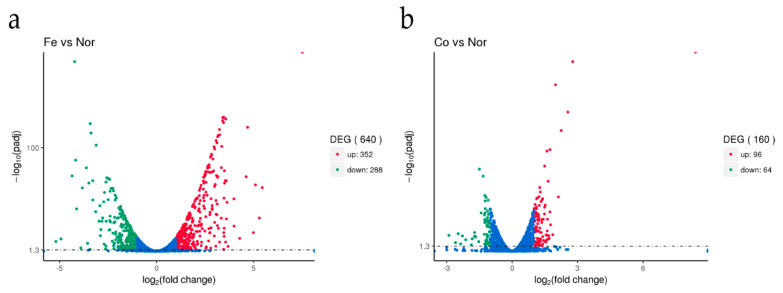
The *S. suis* transcriptome was altered in response to ferrous iron and cobalt toxicity. *S. suis* was grown to an OD_600_ of 0.6, and treated for 15 min with 2 mM FeSO_4_, 0.25 mM CoSO_4_ or deionized water. The figures show the transcription profiles of *S. suis* in the presence of either ferrous iron (**a**) or cobalt (**b**) compared to water. Fe, Co, and Nor in the figures represent ferrous iron, cobalt, and water treatment, respectively. DEG, up, and down represent the differentially expressed genes, up-regulated genes (in red color), and down-regulated genes (in green color), respectively. Genes with a fold change > 2 and an adjusted *p*-value (padj) < 0.05 were defined as differentially expressed genes.

**Figure 2:**
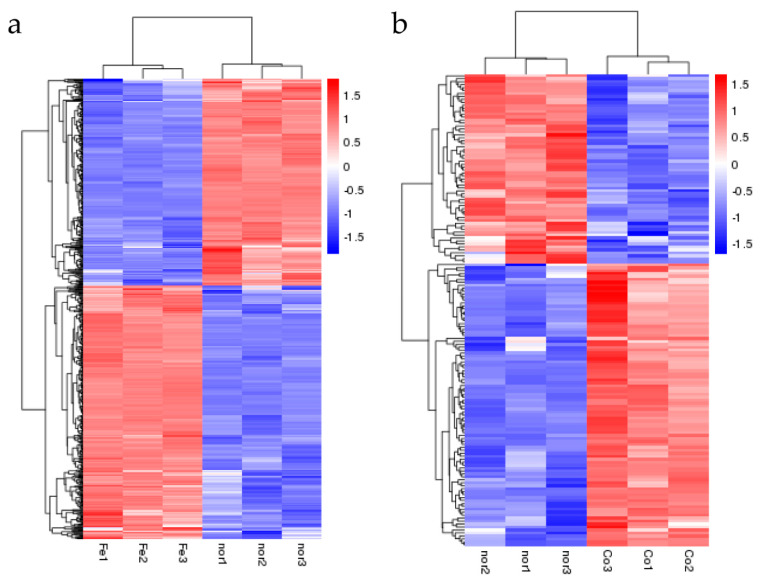
Cluster analyses of the DEGs in response to ferrous iron (**a**) and cobalt (**b**) toxicity based on the FPKM (the expected number of fragments per kilobase of transcript sequence per million base pairs sequenced) values. Fe1-3, Co1-3, and nor1-3 represent the three biological replications of ferrous iron, cobalt, and water treatment, respectively. The color gradient from blue to red represents relative gene expression levels from low to high.

**Figure 3:**
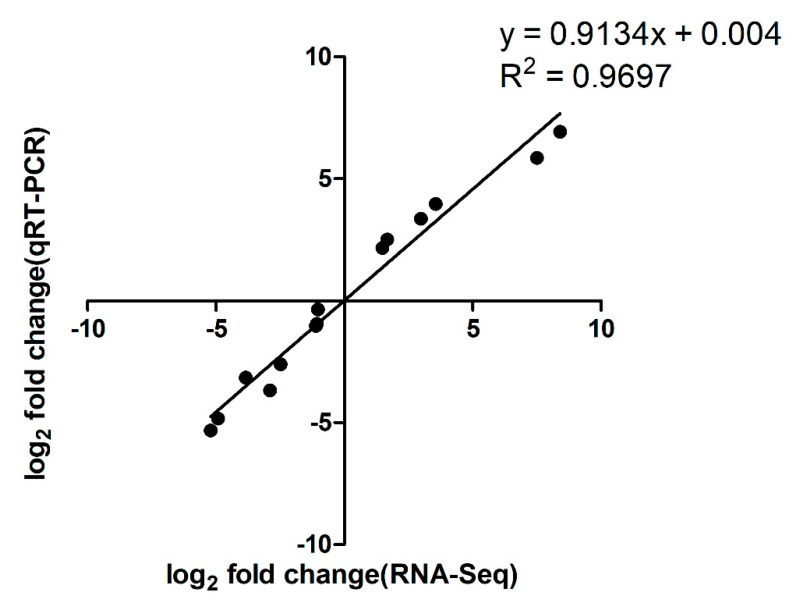
Correlation between RNA sequencing data and qRT-PCR results. A subset of seven genes with varying fold changes was selected to confirm the accuracy of RNA sequencing results by qRT-PCR analysis. The fold changes obtained by these two methods were log_2_ transformed, and the values were plotted against each other to assess their correlations. The fold changes of these genes are shown in Table S5.

After treatment with ferrous iron, a total of 640 genes, i.e., about 30% of the genome, were significantly differentially expressed (Figure 1a and Table S2). Among these, 352 genes were up-regulated and the remaining 288 genes were down-regulated (Figure 1a and Table S2). In the presence of cobalt, only 160 genes were differentially expressed, with 96 genes up-regulated and 64 genes down-regulated (Figure 1b and Table S3). Cluster analyses of the DEGs in response to ferrous iron and cobalt toxicity are shown in Figure 2a,b, respectively. The expression patterns of most of the DEGs among biological replications are similar (Figure 2). As expected, *pmtA*, a gene that has been identified to be ferrous iron and cobalt efflux pump [26], was the most up-regulated gene in the presence of both ferrous iron and cobalt (Tables S2 and S3). Interestingly, most of the DEGs (150 of 160) in response to cobalt toxicity showed the same expression trends in the presence of ferrous iron (Table S4). The accuracy of RNA sequencing results was further confirmed by qRT-PCR analysis. The results showed that the expression of the selected genes was consistent between the two methods (Figure 3 and Table S5).

### 3.3. Gene Ontology (GO) Enrichment Analysis of the DEGs

**Figure 4:**
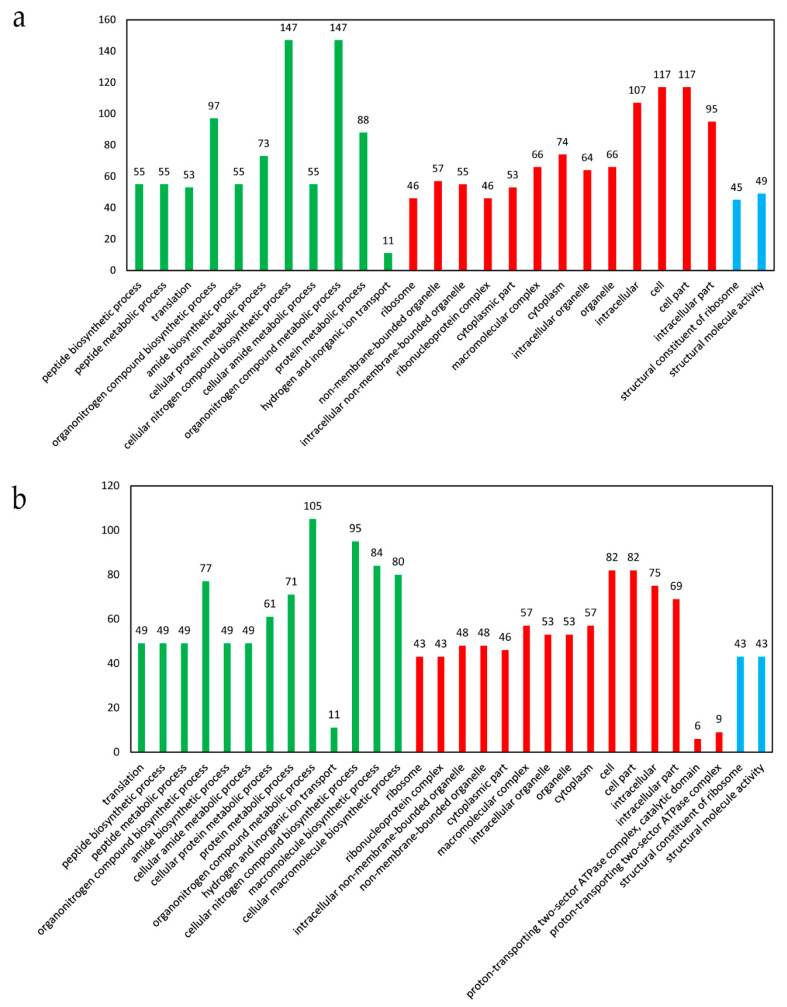
The significantly enriched GO terms of the DEGs in response to ferrous iron toxicity. (**a**) Analysis of all the DEGs. All the significantly enriched GO terms are shown. (**b**) Analysis of the up-regulated genes. The top 30 significantly enriched GO terms are shown. The *X*-axis indicates the enriched GO terms, and the *Y*-axis indicates the number of the DEGs for each GO term. The GO terms in green color belonged to biological processes, those in red belonged to cellular components, and those in blue belonged to molecular function. The GO enrichment analysis was performed using the GOseq method with Wallenius non-central hyper-geometric distribution. The GO terms with a corrected *p*-value < 0.05 were considered to be significantly enriched.

The 640 DEGs identified in the presence of ferrous iron were classified into 1519 GO terms. Among them, 26 terms were significantly enriched, with 11 terms belonging to biological processes, 13 terms belonging to cellular components, and the remaining 2 terms belonging to molecular function (Figure 4a). In biological processes, there were 147 DEGs involved in the cellular nitrogen compound biosynthetic process and organonitrogen compound metabolic process, respectively (Figure 4a). In cellular components, three terms enriched over 100 DEGs, i.e., cell part (117 DEGs), cell (117 DEGs), and intracellular (107 DEGs) (Figure 4a). In molecular function, there were only 49 and 45 DEGs involved in structural molecule activity and structural constituent of ribosome, respectively (Figure 4a). GO enrichment analysis was also performed for the up-regulated and down-regulated genes. The up-regulated genes were significantly enriched into 50 terms, and the top 30 terms are shown in Figure 4b. In contrast, no term was significantly enriched for the down-regulated genes.

**Figure 5:**
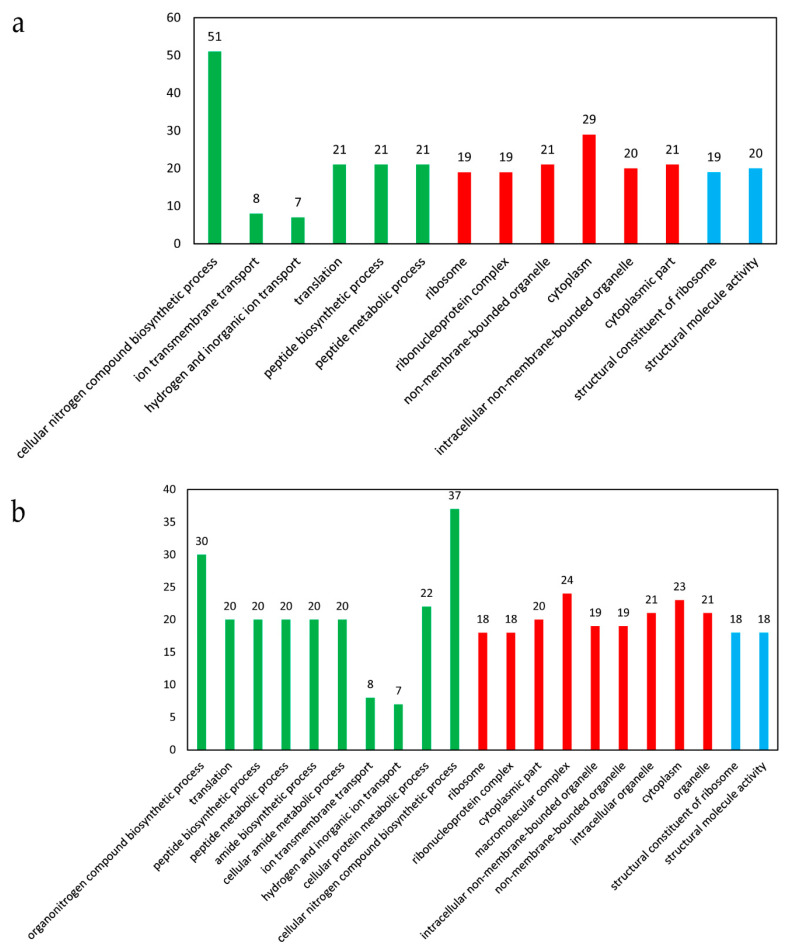
The significantly enriched GO terms of the DEGs in response to cobalt toxicity. (**a**) Analysis of all the DEGs. (**b**) Analysis of the up-regulated genes. The *Y*-axis indicates the enriched GO terms, and the *X*-axis indicates the number of the DEGs for each GO term. The GO terms in a green color belonged to biological processes, those in red belonged to cellular components, and those in blue belonged to molecular function. The GO enrichment analysis was performed using the GOseq method with Wallenius non-central hyper-geometric distribution. The GO terms with a corrected *p*-value < 0.05 were considered to be significantly enriched.

In the presence of cobalt, 14 GO terms were significantly enriched, with 6 terms belonging to biological processes, 6 terms belonging to cellular components, and 2 terms belonging to molecular function (Figure 5a). A total of 51 DEGs were involved in the cellular nitrogen compound biosynthetic process, followed by 29 DEGs in cytoplasm, 21 DEGs in translation, peptide biosynthetic process, peptide metabolic process, non-membrane-bounded organelle, and cytoplasmic part, respectively (Figure 5a). The up-regulated genes could be significantly enriched into 21 GO terms, with 10 terms belonging to biological processes, 9 terms belonging to cellular components, and 2 terms belonging to molecular function (Figure 5b). The down-regulated genes could be significantly enriched into 2 terms belonging to biological processes, i.e., phosphoenolpyruvate-dependent sugar phosphotransferase system (10 genes) and carbohydrate transport (10 genes).

### 3.4. KEGG (the Kyoto Encyclopedia of Genes and Genomes) Pathway Enrichment Analysis of the DEGs

**Figure 6:**
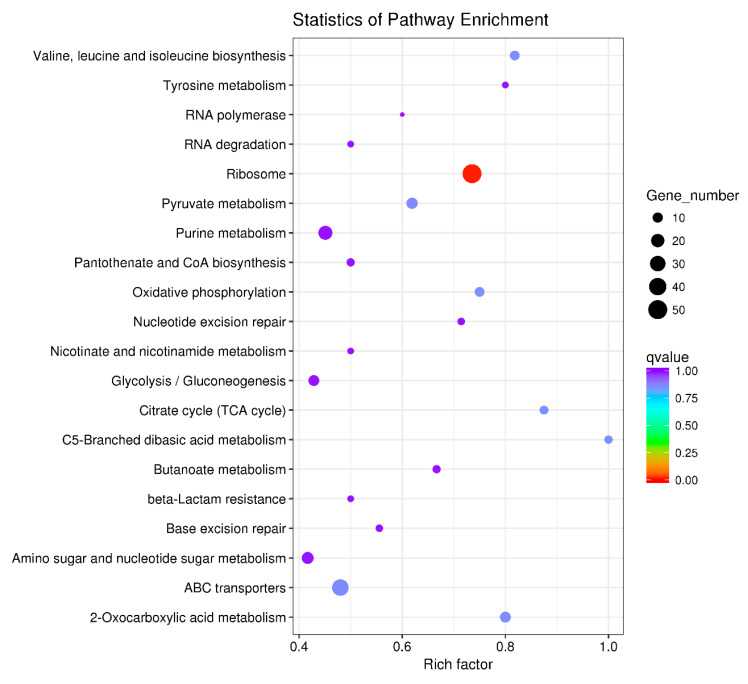
The top 20 KEGG pathways of the DEGs in response to ferrous iron toxicity. The size of the dot indicates the number of the DEGs enriched in the pathway, and the color indicates the *q* value. Rich factor was defined as the ratio of the number of the DEGs enriched in the pathway to the number of all genes annotated to this pathway. *q* value is the corrected *P*-value ranging from 0 to 1, and the lower the *q* value, the greater the pathway enrichment. KEGG pathway enrichment analysis was performed using the KOBAS 2.0 software with the hyper-geometric test. The KEGG pathways with a *q* value < 0.05 were considered to be significantly enriched.

**Figure 7:**
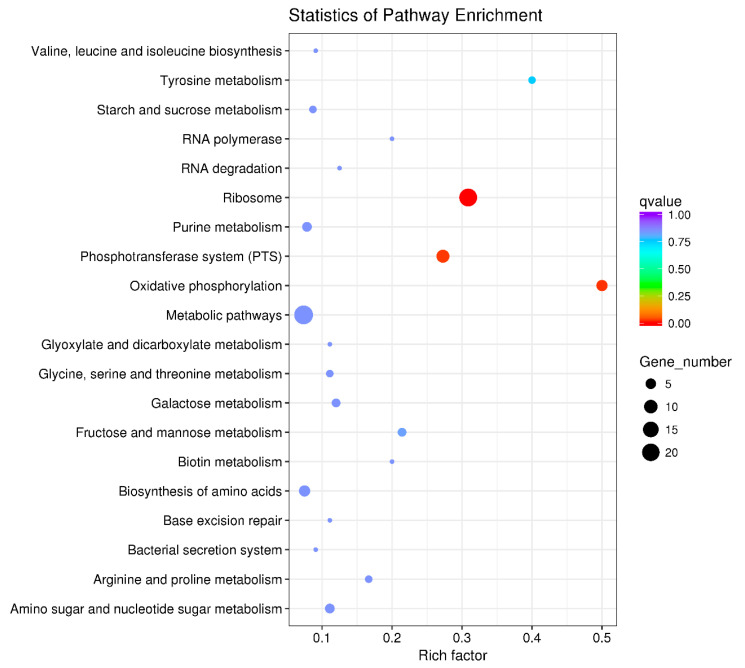
The top 20 KEGG pathways of the DEGs in response to cobalt toxicity. The size of the dot indicates the number of the DEGs enriched in the pathway, and the color indicates the *q* value. Rich factor was defined as the ratio of the number of the DEGs enriched in the pathway to the number of all genes annotated to this pathway. *q* value is the corrected *p*-value ranging from 0 to 1, and the lower the *q* value, the greater the pathway enrichment. KEGG pathway enrichment analysis was performed using the KOBAS 2.0 software with the hyper-geometric test. The KEGG pathways with a *q* value < 0.05 were considered to be significantly enriched.

The 640 DEGs in response to ferrous iron toxicity were enriched into 60 KEGG pathways, and the most enriched 20 pathways are shown in Figure 6. The ribosome pathway was significantly enriched, with a Rich factor of 0.735 (Figure 6). In the presence of cobalt, the 160 DEGs were enriched into 37 pathways, of which the top 20 pathways are shown in Figure 7. Unlike the ferrous iron condition, three pathways were significantly enriched, including ribosome (Rich factor: 0.309), oxidative phosphorylation (Rich factor: 0.500), and the phosphotransferase system (Rich factor: 0.273) (Figure 7).

### 3.5. Several Genes Involved in Oxidative Stress Tolerance Were Significantly Down-Regulated in the Presence of Ferrous Iron

Analysis of the transcriptome results revealed that expression of several genes implicated in oxidative stress tolerance was significantly down-regulated in the presence of ferrous iron (Table 3). In *S. suis*, NADH oxidase has been demonstrated to be involved in resistance to oxidative stress and virulence [35]. Expression of the *nox* gene (SSUSC84_RS03505), which encodes NADH oxidase, was down-regulated approximately 19-fold following treatment with ferrous iron. Thiol peroxidase has been shown to be implicated in response to oxidative stress in *Streptococcus pneumoniae* [36,37]. Treatment with ferrous iron resulted in approximately 18-fold down-regulation of the gene encoding thiol peroxidase (SSUSC84_RS06530). Superoxide dismutase (SodA) is a well-characterized antioxidant enzyme in *S. suis* and other bacterial species [38,39,40,41]. The gene encoding SodA (*sodA*, SSUSC84_RS07245) was down-regulated approximately 14- and 2-fold in the presence of ferrous iron and cobalt, respectively. Rex is a redox-sensing regulator that contributes to oxidative stress response and virulence in *S. suis* [42]. The gene encoding Rex, i.e., SSUSC84_RS05100, was down-regulated approximately 12- and 3-fold following treatment with ferrous iron and cobalt, respectively. Taken together, the expression of certain oxidative stress tolerance-related genes was obviously repressed by ferrous iron, and to a lesser extent, by cobalt.

### 3.6. Treatment with Ferrous Iron and Cobalt Repressed Expression of Several Genes in the Arginine Deiminase System

**Figure 8:**
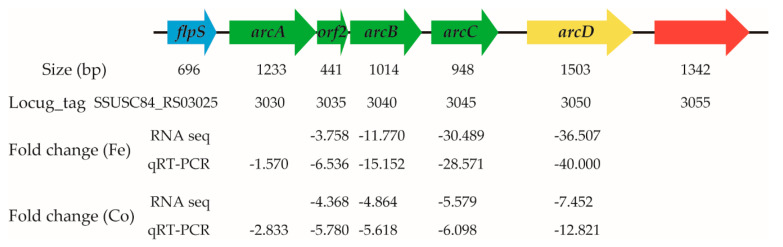
Expression of the genes in the arginine deiminase system after treatment with ferrous iron and cobalt. Expression level of each gene is shown under its locus_tag. The negative values indicate down-regulation. The size for each gene is shown above its locus_tag. The genes *arcA*, *orf2*, *arcB*, and *arcC* form an operon, and encode arginine deiminase, acetyltransferase, ornithine carbamoyltransferase, and carbamate kinase, respectively. The *flpS* and *arcD* genes encode a transcription regulator and arginine-ornithine antiporter, respectively. The qRT-PCR results were analyzed using the unpaired Student’s *t* test, and a *p*-value < 0.05 was considered to be significant.

The arginine deiminase system (ADS) is involved in the metabolism of arginine to ornithine, ammonia, and carbon dioxide, and facilitates biological fitness in streptococcal species [43,44,45,46]. The genes encoding the ADS are commonly organized in an operon-like structure [45]. In *S. suis*, the genes *arcA*, *orf2*, *arcB*, and *arcC* in the ADS form an operon, and these genes encode arginine deiminase, acetyltransferase, ornithine carbamoyltransferase, and carbamate kinase, respectively [45]. Upstream of the operon is the *flps* gene, which encodes a transcription regulator, while downstream of the operon is the *arcD* gene encoding the arginine-ornithine antiporter [45]. The transcriptome data revealed that treatment with ferrous iron resulted in down-regulation of the *orf2*, *arcB*, *arcC*, and *arcD* genes by 3.758-, 11.770-, 30.489-, and 36.507-fold, respectively (Figure 8). After treatment with cobalt, the four genes were down-regulated 4.368-, 4.864-, 5.579-, and 7.452-fold, respectively (Figure 8). Interestingly, although *arcA* was a component of the operon, it was not differentially expressed. Therefore, qRT-PCR analysis, a more sensitive method, was performed to further detect the expression of *arcD* and the genes in the operon. As shown in Figure 8, the results confirmed the accuracy of the expression levels of *orf2*, *arcB*, *arcC*, and *arcD*, and also revealed significant down-regulation of *arcA* in the presence of ferrous iron (fold change, 1.570; *p* < 0.05) and cobalt (fold change, 2.833; *p* < 0.05). Thus, *S. suis* down-regulated expression of several genes in the ADS in response to ferrous iron and cobalt toxicity.

### 3.7. The Genes in an Amino Acid ABC Transporter Operon Were Significantly Up-Regulated in the Presence of Ferrous Iron

**Figure 9:**
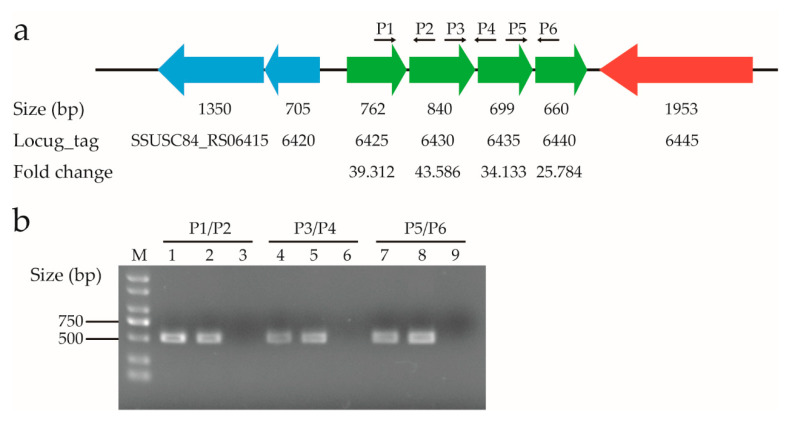
Expression of the genes in an amino acid ABC transporter operon after treatment with ferrous iron. (**a**) Genomic context and expression levels of the genes in the operon. The SSUSC84_RS06425 and SSUSC84_RS06430 genes encode an amino acid ABC transporter ATP-binding protein and glutamine ABC transporter substrate-binding protein, respectively, while the SSUSC84_RS06435 and SSUSC84_RS06440 genes both encode the amino acid ABC transporter permease. The SSUSC84_RS06415 (*vicK*) and SSUSC84_RS06420 (*vicR*) genes encode the VicRK two-component regulatory system, and the SSUSC84_RS06445 gene encodes threonine-tRNA ligase. The expression level of each gene is shown under its locus_tag, while the size of each gene is shown above its locus_tag. P1 to P6 indicate the positions and directions of the primers used for reverse transcription PCR analysis. (**b**) Confirmation of the operon by reverse transcription PCR analysis. The RNA extracted from the *S. suis* SC19 strain was used to synthesize cDNA. Lanes 1, 4, and 7 represent the amplification using cDNA as the template; lanes 2, 5, and 8 represent the amplification using genomic DNA as the template; lanes 3, 6, and 9 represent the amplification using cDNA- (cDNA reaction without reverse transcriptase) as the template. Lane M shows the DL 2000 DNA Marker. The primer pairs used for PCR are shown above the lines, and their sequences are provided in Table S6.

Analysis of the transcriptome data revealed that four adjacent genes, i.e., SSUSC84_RS06425, SSUSC84_RS06430, SSUSC84_RS06435, and SSUSC84_RS06440, were among the top 10 up-regulated genes in response to ferrous iron toxicity (Table S2). After treatment with ferrous iron, they were up-regulated approximately 39-, 44-, 34-, and 26-fold, respectively (Figure 9a). In contrast, none of these genes were included in the DEGs in the presence of cobalt (Table S3). The SSUSC84_RS06425 and SSUSC84_RS06430 genes encode an amino acid ABC transporter ATP-binding protein and glutamine ABC transporter substrate-binding protein, respectively, while the SSUSC84_RS06435 and SSUSC84_RS06440 genes both encode the amino acid ABC transporter permease. The four genes are transcribed in the same direction, with only a few nucleotides separating each other (Figure 9a), leading to the speculation that they are organized in an operon. To test this speculation, reverse transcription PCR analysis was performed with cDNA and the gene-specific primer pairs. For each PCR reaction, the forward primer binding to a specific gene and the reverse primer binding to its adjacent gene were used (Figure 9a). As shown in Figure 9b, all PCR reactions using the cDNA as templates generated products consistent with those generated from genomic DNA, suggesting that these genes were co-transcribed. Together, these four genes form an operon and were significantly up-regulated in the presence of ferrous iron rather than cobalt.

## 4. Discussion

Although the mechanisms underlying resistance to metal toxicity in *S. suis* have been partly elucidated, it was based on studies of individual genes. The global gene transcription profile of *S. suis* in response to metal toxicity has received only limited attention to date [20]. Our finding from a transcriptomic analysis of *S. suis* in the presence of excess metal will undoubtedly provide insights into its response to metal toxicity.

In this study, we explored the transcriptome-level changes in *S. suis* in the presence of excess ferrous iron and cobalt by RNA sequencing. *S. suis* alters the expression of 640 and 160 genes, i.e., approximately 30% and 7.5% of the genome, in response to ferrous iron and cobalt toxicity, respectively. Similarly, a previous study showed that treatment with zinc resulted in differential expression of 117 genes in *S. suis*, of which 71 genes were up-regulated and 46 genes were down-regulated [20]. Since metals are important for many biological processes, the differential expression of so many genes following treatment with metals in *S. suis* was not unexpected. Most of the DEGs in response to cobalt toxicity showed the same expression trends as those in response to ferrous iron toxicity, indicating that the mechanism used by *S. suis* to respond to cobalt was also used to respond to ferrous iron. Consistent with this speculation, PmtA contributes to resistance to both cobalt and ferrous iron toxicity in *S. suis* [26].

The DEGs were subsequently subjected to GO enrichment analysis and KEGG pathway enrichment analysis. The DEGs in response to ferrous iron and cobalt toxicity exhibited generally similar GO terms and KEGG pathways, indicating that excess ferrous iron and cobalt have similar effects on the cellular processes of *S. suis*. However, some differences in the GO terms and KEGG pathways were also observed between the DEGs in the presence of ferrous iron and those in the presence of cobalt, indicating the presence of individual effects for ferrous iron and cobalt. GO enrichment analysis of the up-regulated and down-regulated genes showed that the up-regulated genes in response to ferrous iron and cobalt toxicity both could be significantly enriched into a number of GO terms, while the down-regulated genes could be significantly enriched into none or only a few GO terms. This result suggested that *S. suis* could respond to ferrous iron and cobalt through up-regulation of genes specifically classified into certain GO terms.

The transcriptome data revealed that several genes involved in oxidative stress tolerance were down-regulated in the presence of ferrous iron. Since ferrous iron is reductive, it is expected that *S. suis* down-regulated the expression of the genes implicated in resistance to oxidative stress. In *S. suis* and *Streptococcus pyogenes*, pretreatment with ferrous iron resulted in growth defects of the *pmtA* knock-out strain in the presence of hydrogen peroxide [26,47,48]. Highly reactive hydroxyl radicals generated from the reaction between ferrous iron and hydrogen peroxide have been established to cause toxicity in bacteria [16,49]. In previous studies, growth defects of the *pmtA* knock-out strain under hydrogen peroxide stress were usually attributed to reactive oxygen species [26,47,48]. Our study revealed that down-regulation of the genes involved in oxidative stress tolerance following ferrous iron pretreatment might be another mechanism underlying the growth defects of the mutant.

The expression of *arcA*, a gene in the ADS, was reported to be up-regulated under iron-restricted conditions in *S. suis* [50]. In accordance with this result, our data showed that expression of several genes in the ADS, i.e., *arcA*, *orf2*, *arcB*, *arcC*, and *arcD*, was down-regulated under ferrous iron and cobalt excess conditions. Different from the observations in *S. suis*, a recent study revealed that the expression of the genes involved in arginine catabolism (*arcB*, *arcC*, and *arcD*) was up-regulated in response to ferrous iron toxicity in *S. pyogenes* [48]. Therefore, it is reasonable to speculate that the ADS is involved in response to ferrous iron and cobalt starvation in *S. suis*, and is implicated in resistance to ferrous iron toxicity in *S. pyogenes*.

Analysis of the top 10 up-regulated genes in response to ferrous iron toxicity allowed the identification of an amino acid ABC transporter operon. The transcriptome data also revealed that a number of genes encoding the components of ABC transporters were differentially expressed in response to ferrous iron and cobalt toxicity (Tables S2 and S3). Similarly, in *Enterococcus faecalis*, the genes in response to ferric iron could be significantly enriched into the “amino acid transport and metabolism” COG category [51]. These results together indicate that amino acid transport should play an important role in bacterial response to metal toxicity.

Aconitate hydratase is involved in iron homeostasis and oxidative stress response in certain organisms [52,53,54,55,56]. Consistent with this observation, the gene encoding aconitate hydratase (*acnA*, SSUSC84_RS05690) was one of the most up-regulated genes in response to ferrous iron in *S. suis* (Table S2). Serine protease has been demonstrated to be required for iron homeostasis in mice and humans [57,58,59]. Expression of the gene encoding serine protease (SSUSC84_RS09305) was also up-regulated approximately 24-fold and 3-fold in response to ferrous iron and cobalt, respectively (Tables S2 and S3), indicating the potential role of serine protease in metal homeostasis in *S. suis*.

Although the transcriptome data yielded some interesting findings, they were based solely on bioinformatics analysis. Since the expression of genes in the ADS was significantly down-regulated under ferrous iron and cobalt excess conditions, further studies should be performed to determine whether the ADS plays a role in metal starvation in *S. suis*. Experimental evidence is also needed to examine the involvement of the amino acid ABC transporter operon in resistance to ferrous iron toxicity in *S. suis*. Considering the high expression level of *acnA* in the presence of ferrous iron, one topic of interest would be the function of aconitate hydratase in iron homeostasis, oxidative stress response, and pathogenesis in *S. suis*. Assessments of the role of other top up-regulated or down-regulated genes, such as *sspA*, in metal homeostasis and pathogenesis in *S. suis*, would also be of interest.

In conclusion, multiple genes were differentially expressed in response to ferrous iron and cobalt toxicity in *S. suis*. Most of the DEGs in the presence of cobalt showed the same trends as those in the presence of ferrous iron. Bioinformatics analysis of the DEGs revealed that ferrous iron and cobalt have similar effects on the cellular processes of *S. suis*, and the bacterium could respond to ferrous iron and cobalt toxicity through up-regulation of genes specifically classified into certain GO terms. Expression of genes involved in oxidative stress tolerance was down-regulated in the presence of ferrous iron. Treatment with ferrous iron and cobalt resulted in down-regulation of several genes in the ADS. Furthermore, the genes in an amino acid ABC transporter operon were up-regulated in the presence of ferrous iron.

## Figures and Tables

**Table 1:** Primers used for qRT-PCR analysis.

Gene	Primer	Sequence (5′–3′)	Size (bp)
SSUSC84_RS00185	Q0185F	CTTGAAGGGATGGCTGCTGA	123
	Q0185R	CCTCACTAAAACTGATCCCGGA	
SSUSC84_RS00550	Q0550F	TCGTACGATTGAACAAGCCCA	125
	Q0550R	TAATACGACCGCTGAGACGCT	
SSUSC84_RS01570	Q1570F	CGATGTTGTCCGCAATGTCAC	118
	Q1570R	AACCATCGCTTCTCCTTGTGC	
SSUSC84_RS03030	Q3030F	GAAGCTAACATTCGTGGCCG	112
	Q3030R	GAAGCTAACATTCGTGGCCG	
SSUSC84_RS03035	Q3035F	TCCTTGAGCGTGGTATTGCA	105
	Q3035R	GTCCCGTTGAAACAGGCTCT	
SSUSC84_RS03040	Q3040F	TGGTTTGACAGATGCATGGC	112
	Q3040R	ACGGCCATCACCACAGTAAA	
SSUSC84_RS03045	Q3045R	AGTCGTTGCTTCGCCAAAAC	123
	Q3045F	TCTGCTTCTTGGACAACTGGAAT	
SSUSC84_RS03050	Q3050F	TTCTTCCCGCTCCTTGTTCC	105
	Q3050R	AGTAGAAGCCAAACAGCCGATTT	
SSUSC84_RS06475	Q6475F	TCTTCCGGAACCTTGATGCC	130
	Q6475R	ACGAGGAAGGCTACGCTCTAGC	
SSUSC84_RS07245	Q7245F	AAGCCCAACCTGAACCGAAA	106
	Q7245R	TCAGCAGAATTGGCAGCAGA	
16s RNA	Q16S1	TAGTCCACGCCGTAAACGATG	159
	Q16S2	TAAACCACATGCTCCACCGC	

**Table 2:** Summary of RNA Sequencing data.

Group	Sample	Raw Reads	Clean Reads	Q20(%)	Q30(%)	Mapped Reads	Mapping Ratio (%)
Fe	Fe1	11760266	11516070	97.91	93.85	11364301	98.68%
	Fe2	12974760	12717952	98.12	94.33	12568136	98.82%
	Fe3	11724878	11434962	98.02	94.07	11300186	98.82%
Co	Co1	9204800	8993140	98.01	94.11	8870502	98.64%
	Co2	12938278	12668354	98.09	94.29	12507324	98.73%
	Co3	11516386	11277240	97.92	93.82	11127475	98.67%
Nor	Nor1	11972384	11701188	97.96	93.97	11512748	98.39%
	Nor2	11584884	11384656	98.08	94.28	11203130	98.41%
	Nor3	9594030	9393308	98.26	94.73	9259745	98.58%

**Table 3:** Expression levels of genes involved in oxidative stress tolerance in the presence of ferrous iron and cobalt ^1^.

Gene	Product	Ferrous Iron Treatment		Cobalt Treatment	
		Fold Change	Adjusted *p*-Value	Fold Change	Adjusted *p*-Value
SSUSC84_RS03505	NADH oxidase	−18.734	7.50 × 10^−184^	−1.641	1.63 × 10^−4^
SSUSC84_RS06530	thiol peroxidase	−18.065	8.93 × 10^−89^	−1.690	1.43 × 10^−5^
SSUSC84_RS07245	superoxide dismutase (SodA)	−14.206	6.09 × 10^−62^	−2.044	2.31 × 10^−8^
SSUSC84_RS05100	transcriptional repressor Rex	−12.289	2.52 × 10^−81^	−2.837	1.42 × 10^−21^

^1^ The data are extracted from RNA Sequencing results, and the negative values indicate down-regulation.

## Supplementary Materials

The following are available online at https://www.mdpi.com/2073-4425/11/9/1035/s1. Table S1: The efficiency of each primer pair used for qRT-PCR analysis. Table S2: Summary of the differentially expressed genes in the presence of ferrous iron. Table S3: Summary of the differentially expressed genes in the presence of cobalt. Table S4: Summary of the DEGs that expressed in the same trends in the presence of cobalt and ferrous iron. Table S5. Validation of RNA sequencing results by qRT-PCR analysis. Table S6. Primers used for reverse transcription PCR analysis.
