# Supplementary material for: Transcriptomic Analysis of Streptococcus suis in Response to Ferrous Iron and Cobalt Toxicity
Source: Genes (Basel). 2020 Sep 2;11(9):1035. doi: 10.3390/genes11091035 (PMC7563783; doi:10.3390/genes11091035)
Supplement: Supplementary file 1 [file genes-11-01035-s001.zip › Supplementary Material/Table S1.docx]

**Table S1.** The efficiency of each primer pair used for qRT-PCR analysis.

| **Gene** | **Primer** | **PCR efficiency (*E*) ^1^** |
| --- | --- | --- |
| SSUSC84_RS00185 | Q0185F | 105% |
|  | Q0185R |  |
| SSUSC84_RS00550 | Q0550F | 104% |
|  | Q0550R |  |
| SSUSC84_RS01570 | Q1570F | 104% |
|  | Q1570R |  |
| SSUSC84_RS03030 | Q3030F | 110% |
|  | Q3030R |  |
| SSUSC84_RS03035 | Q3035F | 104% |
|  | Q3035R |  |
| SSUSC84_RS03040 | Q3040F | 107% |
|  | Q3040R |  |
| SSUSC84_RS03045 | Q3045R | 103% |
|  | Q3045F |  |
| SSUSC84_RS03050 | Q3050F | 104% |
|  | Q3050R |  |
| SSUSC84_RS06475 | Q6475F | 103% |
|  | Q6475R |  |
| SSUSC84_RS07245 | Q7245F | 108% |
|  | Q7245R |  |
| 16s RNA | Q16S1 | 101% |
|  | Q16S2 |  |

^1^ The efficiency of each primer pair was determined using serially diluted genomic DNA, as previous described [1]. The efficiency (*E*) is defined as the fraction of target DNA molecules that are copied after one PCR cycle. *E* should be 100% when the number of target DNA molecules is perfectly doubled in every PCR cycle. Several factors, such as primer dimer, might result in *E* > 100%. According to the instructions of the NovoStart SYBR qPCR SuperMix Plus kit, 90% ≤ E ≤ 110% is acceptable for qRT-PCR analysis.

**Reference**

1. Svec, D.; Tichopad, A.; Novosadova, V.; Pfaffl, M.W.; Kubista, M. How good is a PCR efficiency estimate: Recommendations for precise and robust qPCR efficiency assessments. *Biomolecular detection and quantification* **2015**, *3*, 9-16, doi:10.1016/j.bdq.2015.01.005.
