# Supplementary material for: Transcriptomic Analysis of Streptococcus suis in Response to Ferrous Iron and Cobalt Toxicity
Source: Genes (Basel). 2020 Sep 2;11(9):1035. doi: 10.3390/genes11091035 (PMC7563783; doi:10.3390/genes11091035)
Supplement: Supplementary file 1 [file genes-11-01035-s001.zip › Supplementary Material/Table S5.docx]

**Table S5.** Validation of RNA sequencing results by qRT-PCR analysis ^1^.

| **Gene** | **Product** | **Fold change (Fe)** | | **Fold change (Co)** | |
| --- | --- | --- | --- | --- | --- |
|  |  | RNA seq | qRT-PCR | RNA seq | qRT-PCR |
| SSUSC84_RS00185 | MreC | 7.937 | 10.319 | 2.787 | 4.473 |
| SSUSC84_RS00550 | adenylate kinase | 11.783 | 15.619 | 3.190 | 5.702 |
| SSUSC84_RS01570 | PmtA | 182.089 | 57.774 | 338.544 | 122.095 |
| SSUSC84_RS03045 | carbamate kinase | 0.033 | 0.035 | 0.179 | 0.164 |
| SSUSC84_RS03050 | ArcD | 0.027 | 0.025 | 0.134 | 0.078 |
| SSUSC84_RS06475 | CcpA | 0.476 | 0.517 | 0.466 | 0.491 |
| SSUSC84_RS07245 | SodA | 0.070 | 0.112 | 0.489 | 0.780 |

^1^ Fe and Co represent ferrous iron and cobalt treatment, respectively.
