# Supplementary material for: Transcriptomic Analysis of Streptococcus suis in Response to Ferrous Iron and Cobalt Toxicity
Source: Genes (Basel). 2020 Sep 2;11(9):1035. doi: 10.3390/genes11091035 (PMC7563783; doi:10.3390/genes11091035)
Supplement: Supplementary file 1 [file genes-11-01035-s001.zip › Supplementary Material/Table S6.docx]

**Table S6.** Primers used for reverse transcription PCR analysis.

| **Primer** | **Sequence (5’-3’)** | **Size (bp)** |
| --- | --- | --- |
| P1 | ACCCAGAAACTATCGGAGATG | 513 |
| P2 | TTTGCGAGCAATATCAATCTC |  |
| P3 | AACTCTCTGTCTCGCTACGTG | 519 |
| P4 | ATCATGAACTGAACCAAAAGC |  |
| P5 | CCATGCGAATCATCATTTTAC | 522 |
| P6 | TGGAAGACTAAGAAGACGGTG |  |
